# Sensitive detection of lipopolysaccharides by monitoring of interleukin-10 secretion from human PBMCs

**DOI:** 10.17912/micropub.biology.000773

**Published:** 2023-07-05

**Authors:** Daniel Ivanusic, Joachim Denner

**Affiliations:** 1 Sexually transmitted bacterial pathogens and HIV (FG18), Robert Koch Institute, 13353 Berlin, Germany.; 2 Institute of Virology, Department of Veterinary Medicine, Free University Berlin, 14163 Berlin, Germany.

## Abstract

Lipopolysaccharide (LPS) contaminations may falsify immunological experiments and are crucial for pharmaceutical products because they cause life-threatening immune reactions. Here, we present interleukin-10 (IL-10) as a reliable marker to measure LPS contents when the readout of pro-inflammatory cytokines is not favored. This animal free source assay is able to detect LPS with a limit of detection (LOD) of 0.024 EU/ml by monitoring IL-10 secretions from isolated human peripheral blood mononuclear cells (PBMCs).

**Figure 1. PBMCs-based IL-10 assay:**
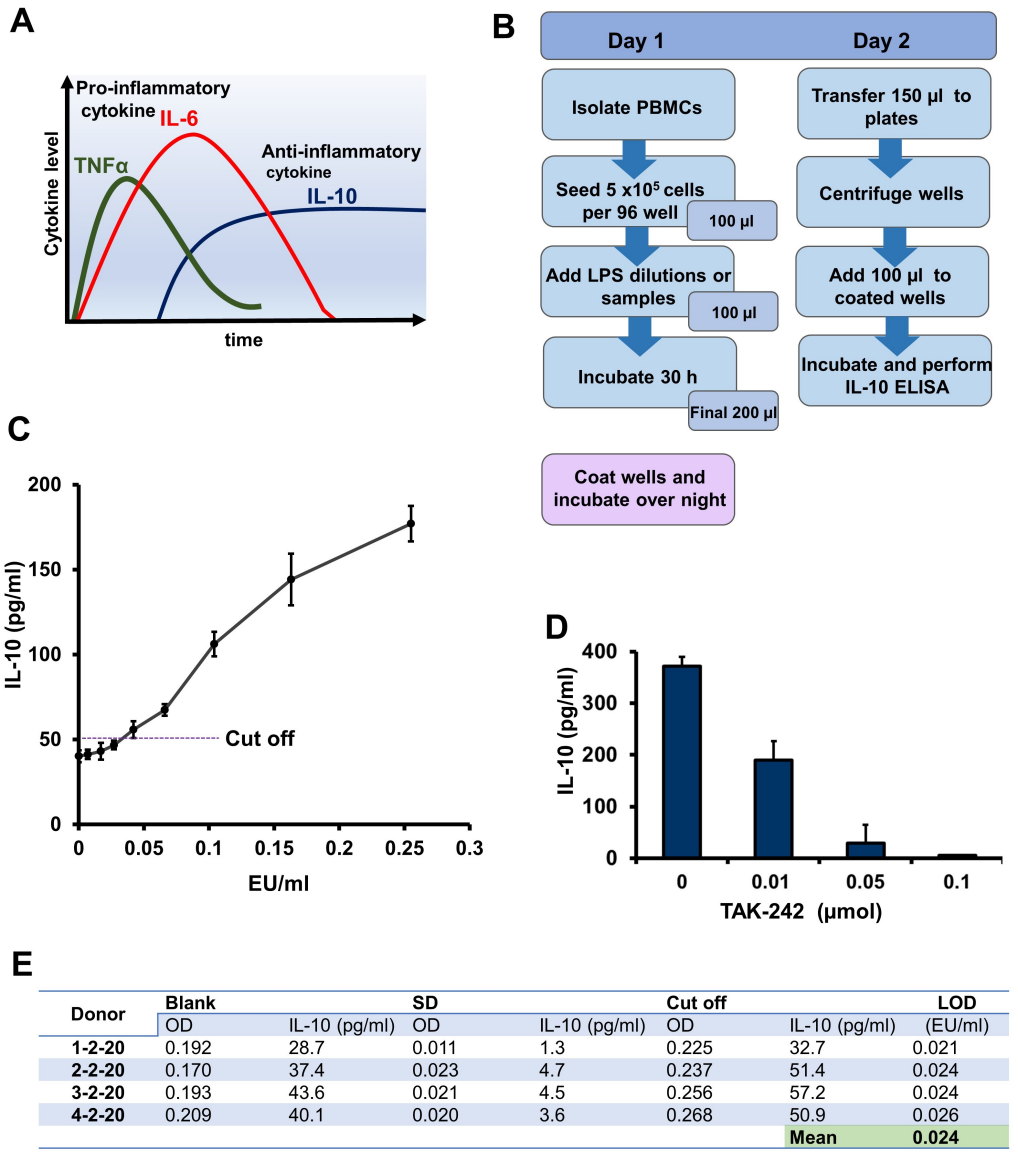
( **A** ) Schematic presentation of pro- and anti-inflammatory cytokine release kinetics. ( **B** ) Workflow diagram for IL-10 release assay. ( **C** ) Graph showing IL-10 secretion values obtained from freshly isolated PBMCs. IL-10 release is dependent from sLPS concentration. ( **D** ) Dose-dependent inhibition of IL-10 secretion by the TAK-242 compound. Freshly isolated PBMCs were incubated with 0.5 EU/ml sLPS and with increasing amounts of TAK-242, IL-10 was monitored by ELISA ( **E** ) Data for calculating limit of detection (LOD) from 4 different donors.

## Description

Contamination of protein preparations with endotoxin when used for immunological experiments, e.g., for testing cytokine release induced by recombinant proteins, may lead to false positive results because endotoxin by itself is a powerful inducer of cytokines. Furthermore, pharmaceutical products should contain only extremely low endotoxin contents in order to avoid life-threatening immune responses, such as hypotension and shock in the patient. Removal of lipopolysaccharide (LPS) from protein preparations is challenging and limited to the removal capacity of each method (Mack et al., 2014) . Therefore, the final protein preparation must be screened for endotoxin content in order to avoid pitfalls during immunological experiments or immunological reactions when contaminated pharmaceutical products were applied. For immunological experiments, it is essential to test for LPS because it cannot be assumed that the control protein contains an equal LPS amount. The content of endotoxin may vary in recombinantly produced proteins, which are under study for their immunological properties.

Endotoxin is a LPS of the outer membrane of most gram-negative bacteria (Wang and Quinn, 2010) . LPS binds first to the LPS-binding protein (LBP) and is transferred to cluster of differentiation 14 (CD14), where myeloid differentiation-2 protein (MD-2) and the Toll-like receptor 4 (TLR4) re-associate. This is considered as an LPS receptor complex (Re and Strominger, 2002; Shimazu et al., 1999; Wright et al., 1989) . The receptor binding leads to a signal transduction involving activation of the transcription factor nuclear factor-kappa B (NF-κB), resulting in the release of cytokines (Chow et al., 1999; Pengal et al., 2006; Rossol et al., 2011) . The limulus amebocyte lysate (LAL) test represents the most commonly used method for quantification of LPS (Reich et al., 2018) . The main component of the LAL test is the hemolymph obtained from wildlife populations of Atlantic horseshoe crabs (Limulus polyphemus). They are collected on beaches and bled to obtain the hemolymph (Maloney et al., 2018) . Despite the huge demand for LPS tests each year, precise numbers of collected *Limulus polyphemus* on the United States east coast are unknown. One third of the blood is taken in order to produce *L. polyphemus* lysates. The mortality rate of horseshoe crabs after bleeding was estimated to be at least 15% to 30% (Maloney et al., 2018) . It is self-evident that it is important to protect this arthropod species with a fossil record extending back to the Ordovician period (approximately 450 million years ago) (Rudkin and Young, 2009) . An alternative pyrogen test is based on monitoring of body temperature increase after injecting the samples to be tested into rabbits. This assay is not limited to the detection of LPS, unlike the LAL test; however, this assay is less sensitive (Richter et al., 1980) . Furthermore, the rabbit pyrogen test is not a quantitative test and shows only a positive or negative pyrogen contamination as the result. In addition, this *in vivo* test is expensive because live animals must be handled in a laboratory facility. Another assay for measuring LPS content is a genetically engineered human TLR4 reporter cell model expressing human TLR4, CD14 and MD-2 (HEK-BlueTM hTLR4) for testing of TLR4 activation. TLR4 activation is assessed by measuring NF-κB-dependent secreted alkaline phosphatase (Govers et al., 2016) . This system is useful for determination of total LPS content in a sample, however the level of cytokine secretion from immune cells cannot be additionally monitored by this method. In the past decade, secretion of IL-6, IL-1β, and tumor necrosis factor alpha (TNF-α) was used for the quantification of pyrogenic contents, such as endotoxins (Schindler et al., 2009) . This assay is called the monocyte activation test (MAT), and it detects pyrogenic material including endotoxins. The cytokines IL-6, IL-1β, and TNF-α belong to the group of pro-inflammatory signals (Turner et al., 2014) . In contrast, IL-10 acts as an anti-inflammatory cytokine and is needed to downregulate pro-inflammatory activities during infection (Conti et al., 2003; Turner et al., 2014) ( Fig. 1A ). The main advantage of the MAT over the LAL assay is that the entity of pyrogen containment can be measured and is therefore not limited to the exclusive detection of LPS.

Our IL-10 LPS assay is based on an overnight incubation of a test substance with isolated peripheral blood mononuclear cells (PBMCs) and quantification of IL-10 values by ELISA ( Fig. 1B ). We isolated PBMCs from four different donors and incubated them with dilutions of standardized LPS (sLPS) to investigate the sensitivity with which the IL-10 secretions induced by LPS can be measured ( Fig. 1C ). Our results showed that the assay is able to reach a mean limit of detection (LOD) of 0.024 EU/ml for LPS ( Fig. 1E ). MAT assays measuring IL-6 as the readout can reach a LOD between 0.04 EU/ml (Etna et al., 2020) and 0.004 EU/ml (Solati et al., 2022) . This means that the measurement of IL-10 secretions with a LOD of 0.024 is an alternative marker to quantify pyrogenic content. An IL-10 release value induced by a sLPS dose of 0.5 EU/ml could be inhibited by adding the TLR4 inhibitor TAK-242, a small-molecule-specific inhibitor of TLR4 signaling ( Fig. 1D ). TAK-242 abrogates the secretion of LPS-induced inflammatory cytokines by binding to the intracellular domain of TLR4 and does not interact with LPS directly (Matsunaga et al., 2011) . The IL-10 response to LPS is mediated by TLR4 activation; therefore, pre-incubation of PBMCs with TAK-242 for 1 h before adding sLPS abrogated secretions of IL-10 in a dose-dependent manner as expected ( Fig. 1D ). This result means that the measured IL-10 released from cells in our newly developed assay is caused specifically by TLR4 activation. A critical point during monitoring of IL-10 release is the use of culture media supplemented with fetal calf serum (FCS). FCS may also be a source of LPS. There are reports that FCS is contaminated with pyrogenic substances (Kirikae et al., 1997; Rinehart and Keville, 1997; Shiigi and Mishell, 1975) and obviously different batches contain different amounts of such contaminants. It is important that for performing an IL-10 assay, all used material should be free of pyrogenic contaminants. To achieve this, different lots of FCS should be screened for low LPS content; however, to avoid time-consuming work to exclude a potential pyrogenic contamination source, we used serum-free media for PBMC cultivation. Serum-free media are standardized and contain an extremely low amount of trace pyrogenic substances. There is also the necessity to test new charges of consumables to make sure that they do not produce a background higher than 30–50 pg/ml IL-10 when PBMCs were incubated without sLPS. A higher background will be answered by a lower detection limit. Consumable material, like cell culture plastic, can be also contaminated with pyrogens and must be tested before use.

In summary, we showed that the readout of secreted IL-10 is suitable to use for the quantification of pyrogens, such as LPS, and is comparable to other sensitive methods, such as the LAL assay. The estimated LOD was 0.024 EU/ml. However, there are potential limitation of the novel assay: proteins which are intrinsically immunosuppressive or which are modulating the cytokine release by TLR4 activation will falsify this assay. There are reports, that the elements platinum, nickel, cobalt and the compound cisplatin activate pathways downstream of TLR4 to a similar extent as the known for LPS (Babolmorad et al., 2021; Oblak et al., 2015) . In addition, taxol (paclitaxel) and viral glycoproteins can also induce cytokine expression by TLR4 activation (Halajian et al., 2022; Rajput et al., 2013) .

**Ethical statement**

The use of human blood has been approved by the ethical commission at the Medical Faculty of Humboldt University, Berlin. Written informed consent was provided by study participants.

## Methods

**PBMCs isolation and cell culture**

PBMCs were isolated from the blood of healthy donors by Ficoll-Hypaque (PAA Laboratories, Linz, Austria), a density centrifugation using Leucosep tubes (Greiner Bio-one GmbH, Frickenhausen, Germany). Cells were washed and dissolved in cell culture medium (CD 293, Gibco Medium for suspension cells, Thermo Fischer, Langenselbold, Germany) and supplemented with 100 IU/mL penicillin, 100 μg/mL streptomycin (PAA Laboratories, Cölbe, Germany), and 2 mM L-glutamine (Biochrom AG, Berlin, Germany).

**IL-10 ELISA**

Release of IL-10 was quantified by the Human IL-10 ELISA Set (BD Biosciences, San Diego, USA). For blocking of coated 96-well ELISA plates (NucMaxiSorp, ThermoFisher Scientific, Langenselbold, Germany), we used a FCS (Biochrom AG, Berlin, Germany) batch, which induced a low background of IL-10 values. 5 x 10 ^5^ of isolated PBMCs in 100 μl serum-free cell culture medium for suspension cells (Gibco CD 293) were placed in a 96 well and incubated with 100 μl of a dilution of an LPS standard (sLPS) in culture medium, using a stock sLPS containing 35 EU/ml taken from the Pierce LAL Chromogenic Endotoxin Quantitation Kit (Thermo Scientific, Braunschweig, Germany). The cells were incubated at 37 °C for 30 h. 150 μl of supernatant was carefully transferred to a 96-well plate and centrifuged at 2,000 g for 10 min. 100 μl of supernatant were then transferred to coated ELISA plates. All other steps performing IL-10 ELISA were followed accordingly to manufactures instructions (BD Biosciences, San Diego, USA). TAK-242 was purchased from Selleck Chemicals (Shanghai, China) and dissolved in dimethyl sulfoxide (DMSO) (Carl Roth, Karlsruhe, Germany). From this stock, we prepared dilutions that we used in our assay. The cut-off was calculated as the mean IL-10 release value of without sLPS plus three standard deviations (SD).
